# The Gut Microbiota-Related Antihyperglycemic Effect of Metformin

**DOI:** 10.3390/ph18010055

**Published:** 2025-01-06

**Authors:** Izabela Szymczak-Pajor, Józef Drzewoski, Małgorzata Kozłowska, Jan Krekora, Agnieszka Śliwińska

**Affiliations:** 1 Department of Nucleic Acid Biochemistry, Medical University of Lodz, 251 Pomorska Str., 92-213 Lodz, Poland; malgorzata.kozlowska@umed.lodz.pl; 2 Central Teaching Hospital of the Medical University of Lodz, 251 Pomorska Str., 92-213 Lodz, Poland; jozef.drzewoski@umed.lodz.pl (J.D.); j.krekora@csk.umed.pl (J.K.)

**Keywords:** glucose-lowering effect, insulin resistance (IR), metformin, microbiota, type 2 diabetes mellitus (T2DM)

## Abstract

It is critical to sustain the diversity of the microbiota to maintain host homeostasis and health. Growing evidence indicates that changes in gut microbial biodiversity may be associated with the development of several pathologies, including type 2 diabetes mellitus (T2DM). Metformin is still the first-line drug for treatment of T2DM unless there are contra-indications. The drug primarily inhibits hepatic gluconeogenesis and increases the sensitivity of target cells (hepatocytes, adipocytes and myocytes) to insulin; however, increasing evidence suggests that it may also influence the gut. As T2DM patients exhibit gut dysbiosis, the intestinal microbiome has gained interest as a key target for metabolic diseases. Interestingly, changes in the gut microbiome were also observed in T2DM patients treated with metformin compared to those who were not. Therefore, the aim of this review is to present the current state of knowledge regarding the association of the gut microbiome with the antihyperglycemic effect of metformin. Numerous studies indicate that the reduction in glucose concentration observed in T2DM patients treated with metformin is due in part to changes in the biodiversity of the gut microbiota. These changes contribute to improved intestinal barrier integrity, increased production of short-chain fatty acids (SCFAs), regulation of bile acid metabolism, and enhanced glucose absorption. Therefore, in addition to the well-recognized reduction of gluconeogenesis, metformin also appears to exert its glucose-lowering effect by influencing gut microbiome biodiversity. However, we are only beginning to understand how metformin acts on specific microorganisms in the intestine, and further research is needed to understand its role in regulating glucose metabolism, including the impact of this remarkable drug on specific microorganisms in the gut.

## 1. Introduction

Diabetes, especially type 2 diabetes mellitus (T2DM) is one of the most common chronic diseases in the world. According to the International Diabetes Federation Atlas, diabetes affects approximately 640 million people globally, and it is predicted that by 2045 this number will increase to over 783 million [1]. T2DM is characterized by hyperglycemia resulting from the combination of impaired regulation of glucose metabolism by insulin, known as insulin resistance (IR), and reduced insulin secretion caused by pancreatic islet β-cell dysfunction [2,3,4,5,6]. However, the pathogenesis of T2DM is still not fully understood [7,8]. Literature data attribute the development of T2DM to both genetic and environmental factors, in particular the lack of physical activity and the Western diet [9]. Interestingly, numerous studies have shown adverse changes in both the function and composition of the gut microbiome in patients with T2DM. A major feature of the intestinal dysbiosis observed in patients suffering from hyperglycemia associated with T2DM is the reduction of butyrate-producing bacteria (i.e., *Butyricinomas* spp., *Allobaculum*, *Subdoligranulum*, *Roseburia intestinalis*, *Roseburia inulinivorans*, *Clostridium species*, *Faecalibacterium prausnitzii*, *Eubacterium rectale*) and the increase in opportunistic pathogens such as *Clostridium hathewayi*, *Clostridium symbiosum* [10]. However, emerging evidence suggests an important role for the gut microbiome in the prevention, treatment, and glycemic control of T2DM [11].

Metformin, a biguanide derivative, is still recommended as a first-line drug in the pharmacotherapy of T2DM, unless it is contraindicated; this is due to, inter alia, its general availability, significant reduction in glucose levels, safety and low price [10]. Interestingly, despite the fact that metformin has been used for over 60 years, its mechanisms of action are still under intensive investigation. Initially, the main site of metformin action was believed to be the liver, where the drug controls glucose production. However, since intravenous administration of metformin was observed to lower glucose levels to a lesser extent than after oral administration, the gastrointestinal system gained the attention of researchers and clinicians [12]. McCreight et al. report that plasma concentrations of metformin are 30–300 times lower than those in the jejunum [13]. Furthermore, it has been shown that the drug suppresses the absorption of glucose from the gastrointestinal tract in both rodents and people with T2DM [14,15,16,17]. It has therefore been suggested that, in addition to the liver, the intestine is another target of metformin activities, which may account for its glucose-lowering effect [10]. It has also been proposed that by affecting the composition of gut microflora, metformin exerts an antihyperglycemic effect [13]. It is worth underlining the fact that Cabreiro et al. were the first to report that metformin extended the lifespan of *Caenorhabditis elegans* cocultured with *E. coli* (as a food source), by altering *E. coli* metabolism [18]. Active bacterial metabolism is a key nutritional requirement for *C. elegans*. The disturbances of active bacterial metabolism delay development and extend lifespan of the worm. Metformin causes not only 33% elevation in S-adenosylhomocysteine (SAH) level, but also an 86% increase in S-adenosylmethionine (SAMe), which inhibits the folate cycle and reduces methionine production by blocking methylenetetrahydrofolate reductase, leading to increased *C. elegans* viability [18,19]. Further research is necessary to better understand the mechanism by which metformin exerts its antiaging effect. The molecular mechanisms of the multidirectional action of metformin are also the subject of research conducted on rodents. The results of these studies have shown that the drug can protect against the development of cardiovascular complications already at the stage of impaired glucose tolerance [20], and weaken the progression of chronic kidney disease, regardless of the presence of diabetes [21], but that it also protects the kidneys and liver against the negative effects of diabetes [22]. Interestingly, metformin-induced changes in the intestinal microbiota diversity were found to not only reduce blood glucose level, but also to be associated with some adverse effects, such as folic acid deficiency and gastrointestinal disorders including nausea, vomiting, flatulence and constipation [18,23]. The complex mechanism of action of metformin on the gastrointestinal tract and its bacterial flora and the consequences of these actions are not fully explained. Therefore, the purpose of our review was to provide an overview of the current state of knowledge regarding the relationship between the effects of metformin on the microbiome and the resulting reduction in blood glucose concentration.

## 2. Search Methodology

To sum up the current scientific literature regarding gut microbiota-related antihyperglycemic effect of metformin, Google Scholar, PUBMED and the university library were searched to find the relevant articles published between 1975 and 2024. The following combinations of keywords were used: Metformin OR biguanide AND microbiota OR gut microbiota OR human gut microbiota OR altered gut microbiome AND short chain fatty acids OR gut permeability OR low grade inflammation OR sub-inflammation OR bile acids circulation. Finally, in vitro, animal and human studies, including clinical trials were included into review.

## 3. Physiological Gut Microbiota: Composition and Functions

### 3.1. Composition of Human Gut Microbiota

The human body is colonized by trillions of microbial cells [24,25]. This number is tenfold greater than those of somatic and germ cells, being approximately 1 × 10^14^ compared to 1 × 10^13^ of eukaryotic cells [26]. The human microbiota are composed of various bacteria, eukaryotic viruses, fungi and bacteriophages that inhabit both body surfaces and cavities and ensure homeostasis of the microbiome [27]. Notably, the composition and activity of the microbiota is closely related to health and the development of disease [28]. The biodiversity of human microbiota is influenced by lifestyle, type of birth delivery, medications, diet in the first years of life, and host genetics. It comprises approximately 1000 species, among which the main component is made up of bacteria belonging to the five main phyla: *Firmicutes*, *Bacteroidetes*, *Proteobacteria*, *Actinobacteria* and *Verrucomicrobia* [29]. These microorganisms inhabit nearly every surface of the human body and colonize several organs in the human genitourinary, gastrointestinal and respiratory tracts [30,31]. However, it should be emphasized that most microorganisms of the microbiota occur in the digestive tract, especially in the colon [27]. The microbiota colonize the surface of the intestinal mucosa, which is approximately 200–300 m^2^ [25,32,33,34]. It has been suggested that the intestinal surface is rich in nutrients that favor the existence of approximately 100 trillion microorganisms. These microbes encode 100 times more genes than the human genome [24,35].

Literature data indicate that the human gut microbiome includes at least 1800 genera and 15,000–36,000 species of bacteria. These microorganisms have evolved in symbiosis with their host. More than 70% of these microbes (10^11^–10^12^ cells/mL) inhabit the colon [36]. In turn, the small intestine is settled by approximately 10^3^–10^9^ cells/mL of microorganisms [37]. These large numbers of gut bacteria belong mainly to nine phyla: *Actinobacteria*, *Firmicutes*, *Fusobacteria*, *Proteobacteria*, *Spirochaetes*, *Bacteroidetes*, *Verrucomicrobia*, *Cyanobacteria*, and *VadinBE97*. Among these phyla, most species belong to the *Proteobacteria*, *Bacteroidetes*, *Firmicutes* and *Actinobacteria* [38].

The microflora of the gastrointestinal tract is commonly divided into transient flora (allochthonous flora) and indigenous flora (autochthonous flora). Autochthonous flora include resident microbes that settle specific habitats in the gastrointestinal tract. These microorganisms participate in the absorption of nutrients occurring in the intestinal mucosa. In turn, transient/allochthonous microbes cannot colonize specific places; they are usually found in the central lumen and constitute a part of the fecal stream [39]. The autochthonous gut microflora is mainly composed of two anaerobic bacterial phyla, *Firmicutes* and *Bacteroidetes*. Less-represented taxa also include *Methanobrevibacter smithii* [40]. Among the less represented taxa are *Actinobacteria*, *Proteobacteria*, *Verrucomicrobia* and *Fusobacteria* [40]. It is believed that the allochthonous microbiota possess specialized mechanisms that are harmless to the host and protect the host organism from endogenous antimicrobial peptides. Thus, the allochthonous gut microbiota evolve in symbiosis with the host [41]. The overall homeostasis of the intestinal microbiome is influenced by the interaction of many factors, including the translocation, migration, competition, elimination and reproduction rates of the microbiota, as defined by regional growth conditions [42,43]. The composition of each individual’s microbiome is unique, and this feature becomes more and more evident with the age of the host and the influence of environmental and demographic factors [32,44,45,46]. Increasing evidence shows that these microorganisms live in harmony with their host. In turn, the occurrence of disturbances in the composition and the integrity of the intestinal microbiome adversely affects health, and may increase susceptibility to various diseases and accelerate their progression. [44,47].

### 3.2. Microbiota Functions

**Figure 1:**
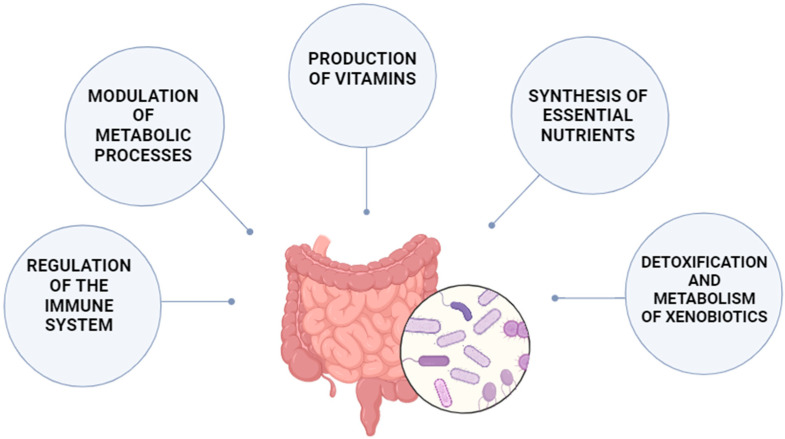
Microbiota functions.

Growing evidence has shown that gut microbiota play an important role in numerous biological processes in humans. The intestinal microbiota participate in metabolism, nutrient extraction and immune processes [48]. The microbiota exerts an effect on biological processes by several mechanisms. Firstly, they are a source of metabolic genes which provide independent enzymes and biochemical pathways engaged in energy and nutrient extraction from food [49]. Secondly, the gut microbiota synthesize numerous molecules, including amino acids, vitamins, short-chain fatty acids (SCFAs) and lipids [50]. Finally, the microbiota protect the host from external pathogens by producing antimicrobial substances and play a crucial role in the development of the intestinal mucosa and immune system. Thus, the human microbiota can be regarded as an active metabolic organ that supports key functions in the host, including among others, the modulation of metabolic processes, neurotransmitter production, nutrient extraction, detoxication and xenobiotic metabolism, synthesis of essential nutrients and regulation of immune system [27,51]. It has been shown that maintaining the biodiversity of a functionally stable microbiome favors the health of its host [52]. Microbiota functions are summarized in Figure 1. In turn, changes in the composition of the human microbiota that dysregulate the symbiotic interplay between host and its microorganisms, known as dysbiosis, are suggested to be involved in the development of numerous diseases, including obesity, T2DM, autoimmune and neurological diseases, and cancers [53,54].

Dysbiosis has been categorized into three states: the reduction in beneficial microorganisms, the elevation of opportunistic pathogenic microorganisms, and the decrease in microbiota diversity. It is worth emphasizing, however, despite this classification, these three phenomena usually occur simultaneously [55]. Therefore, factors promoting the dysbiosis of microbiota are suggested as potential risk factors for the development of the dysbiosis-related diseases mentioned above [55]. As such, any factor that at least partially restores the microfloral balance could be used to treat or prevent the adverse consequences of dysbiosis. Interestingly, a growing body of evidence suggests that metformin, an older-generation antihyperglycemic drug, can partially restore the balance of gut microflora [56,57,58].

Gut microbiota-derived SCFAs improve insulin sensitivity in peripheral tissues, such as the liver, skeletal muscle and adipose tissue. SCFAs are produced in the gastrointestinal tract primarily by intestinal microbial anaerobic fermentation and, to a lesser extent, by fermentation of undigested dietary fiber derived from plant foods. Fermentation of proteins and nucleic acids only represent a minimal source of SCFAs. These compounds serve important metabolic functions, and are crucial for intestinal health [59].

Three main SCFAs control energy supply, metabolism, and the homeostasis of the intestinal microenvironment: acetate, propionate and butyrate. Acetate, produced by *Bifidobacteria*, *Bacteroidetes* and *Lactobacillus*, is an important substrate for the synthesis of cholesterol and fatty acids in both the liver and in other tissues. In turn, propionate produced by bacteria, i.e., *Propionibacterium* sp., *Clostridium* sp., *Megasphaera* sp., *Propionibacterium shermanii*, *Bacteroides species* (i.e., *Bacteroides fragilis* and *Bacteroides eggerthii*), *Veillonella* spp. and *Acidaminococcus* spp., upregulates the expression of the gene encoding leptin, thus protecting against the development of diet-induced obesity. Leptin is a hormone that reduces appetite and feelings of hunger. Butyrate is mostly produced by bacteria belonging to the *Clostridium* cluster of the phylum *Firmicutes,* i.e., *Eubacterium*, *Subdoligranulum*, *Faecalibacterium*, *Coprococcus*, *Anaerostipes*, *Roseburia* and *Anaerobutyricum*. Acetate exerts lipogenic effects, while propionate tends to be gluconeogenic. Butyrate not only participates in the regulation of cell proliferation and differentiation, homeostasis of colonic mucosal and enhancement of insulin sensitivity, but also reduces oxidative stress, protecting colonocyte membrane function and increasing energy expenditure [60,61,62].

The role of SCFAs secreted by the gut microbiota on metabolic disease have attracted the interest of clinicians and researchers [63]. The beneficial effects of SCAFs on glucose metabolism may be exerted in many ways. One is the stimulation of intestinal hormone receptors, especially free fatty acid receptor 2 (FFAR2), known as G protein-coupled receptor 43 (GPR-43), and free fatty acid receptor 3 (FFAR3), known as (GPR-41) [63,64,65]. FFAR2 and FFAR3 are expressed on enteroendocrine L cells. Binding SCAFs to FFAR2 and FFAR3 activates the secretion of glucagon-like peptide-1 (GLP-1) and peptide YY; GLP-1 regulates insulin secretion by pancreatic β-cells and glucose metabolism [66]. Notably, the beneficial effects of butyrate and propionate on energy homeostasis and glucose signaling, which activate intestinal gluconeogenesis, have been observed in animal studies [67].

The goblet cells in the epithelium of the gastrointestinal tract synthesize a thick mucus gel that covers its surface. This layer of intestinal mucus provides a habitat for intestinal microflora: mucus-associated microorganisms settle the mucus layer by attaching to mucin glycans, which further promotes their development. Interestingly, some bacteria affect the secretion of mucus; in particular, *Akkermansia muciniphila* inhabits the mucus layer in the human intestine, where it breaks down mucin, which additionally stimulates its production. In addition, intestinal mucus serves as a barrier to protect the host against pathogenic microorganisms and their components, i.e., lipopolysaccharide (LPS) derived from Gram-negative bacteria [68,69]. Numerous studies have shown that in some metabolic disorders, LPS concentrations in the intestine and plasma increase as a result of increased intestinal barrier permeability. This adverse response has been proposed as a major factor contributing to the development of chronic inflammation, which is associated with insulin resistance and the development of obesity and T2DM [70,71,72,73]. Thus, the maintenance of the intestinal barrier integrity, which depends on the biodiversity of gut microbiota, plays a key role in the development and progression of metabolic diseases. It is known that the mucus layer produced by enterocytes maintains normal gastrointestinal functions and gut permeability by providing substrates for gut bacterial growth, protection and adhesion [74,75,76]. There is growing evidence that colonization of the gut microbiota, especially by Gram-negative bacteria such as the *Enterobacteriaceae* on the mucus layer, is responsible for high-fat diet (HFD)-mediated development of gut dysbiosis and the further development of metabolic disorders, including diabetes and obesity in mice [77,78,79]. Cani et al. report that changes in the composition of the gut microbiota were associated with increased serum LPS levels and intestinal permeability, and reduced glucose tolerance in HFD-fed mice [71].

Bile acids (BAs), steroid-based amphipathic molecules, promote the absorption of lipids and fat-soluble vitamins and also act as emulsifiers to aid the digestion of lipids [80]. The synthesis of BAs occurs in the liver, primarily in the periventricular hepatocytes, liver cells surrounding the central hepatic vein [81]. Two of the primary bile acids, i.e., those directly formed in the liver, are cholic acid and chenocholic acid [82]. The synthesized BAs are secreted by hepatocytes and transported to the gallbladder by the biliary ducts. In turn, postprandial gallbladder contraction results in BA release to the duodenum [83,84]. In the intestine, bacterial flora convert primary BAs into secondary BAs such as deoxycholic acid, lithocholic acid and derivatives of cholic acid and chenodeoxycholic acid, through a series of deconjugation or dihydroxylation modifications [83,85]. Bacterial bile salt hydrolase (bsh) catalyzes the deconjugation of taurine- and glycine-conjugated BA, leading to elevated level of unconjugated BAs. Deconjugation of primary BAs by gut microflora increases the heterogeneity of this group of molecules [56].

After the BAs are actively absorbed in the terminal ileum, they are transported back to the liver via the portal vein. They are reconjugated in the liver and excreted again, together with newly-synthesized BAs. Additionally, a small fraction of BAs leaves this enterohepatic circulation, allowing these molecules to either reach the systemic circulation or to be excreted in the feces [83,85]. The BA fraction from the systemic circulation regulates numerous physiological processes, i.e., glucose and lipid homeostasis, inflammation, intestinal motility and energy expenditure, as well as the growth and configuration of gut microbiota [85]. BAs act as signaling molecules, as they bind and activate the G-protein-coupled receptor (TGR5) and the nuclear farnesoid X receptor (FXR) [86]. BAs-FXR signaling increases the synthesis of glucagon and suppresses the gluconeogenesis by the inhibition of G6Pase, carbohydrate-responsive element-binding protein (ChREBP) and phosphoenolpyruvate carboxykinase (PEPCK) in the liver. BAs-FXR signaling also reduces the production of GLP-1, whereas BAs-TGR5 signaling triggers the expression and secretion of GLP-1 in the intestinal L cells [85]. Physiologically, after consuming food, GLUT-2 and sodium-glucose cotransporter-1 (SGLT-1) in the upper small intestine transports glucose from the intestine’s lumen into enterocytes. GLP-1 is released from the intestinal L cells in response to nutrient intake, particularly carbohydrates and fats [87,88,89,90,91]. GLP-1 delays stomach emptying, which slows the rate at which glucose enters the bloodstream and regulates glucose metabolism by inhibiting glucagon secretion from pancreatic α-cells. Its suppression helps lower blood glucose levels post-meal. In the presence of elevated blood glucose, GLP-1 enhances the release of insulin from pancreatic β-cells. In turn, in the pancreatic β-cells, insulin production is stimulated by both BAs-FXR and BAs-TGR5 signaling. Additionally, TGR5-BAs signaling stimulates glucose-induced secretion of insulin in pancreatic α-cells, leading to transformation of pro-glucagon to GLP-1 and secretion of GLP-1. BAs-TGR5 signaling also increases energy expenditure by stimulating the conversion of inactive thyroxine (T4) into active tri-iodothyronine (T3) in brown adipose tissue and skeletal muscles [85]. It has been also reported that BAs-FXR signaling regulates lipid metabolism. Insulin and glucose activate glycolysis, the process leading to the production of acetyl coenzyme A (acetyl-CoA). Acetyl-CoA, a key molecule that participates in many biochemical reactions in protein, carbohydrate and lipid metabolism, is also a precursor of various fatty acids and cholesterol. In turn, activated FXR/ small heterodimer partner (SHP) signaling suppresses the steroid response element-binding protein 1c (SREBP-1c), leading to the inhibition of lipogenesis. SREBP-1c activates genes engaged in lipogenesis such as fatty acid synthase (FAS), acetyl CoA carboxylase (ACC), and stearoyl CoA desaturase (SCD) [92].

## 4. Gut Dysbiosis Is Associated with Insulin Resistance

Numerous studies have shown a reduction in Firmicutes and butyrate-producing bacterial species, including *Eubacterium rectale*, *Clostridium* spp. (i.e., *Clostridium coccoides*, *Clostridium leptum*), *Roseburia intestinalis*, *Faecalibacterium prausnitzii* and *Roseburia inulinivorans* in T2DM patients. Interestingly, it has also been observed that T2DM favors the occurrence of *Fusobacterium*, *Ruminococcus*, and *Blautia* genera, whereas the number of *Bacteroides*, *Akkermansia*, *Bifidobacterium*, *Faecalibacterium* and *Roseburia* genera negatively correlates with the disease [93,94,95,96,97].

Physiologically, the intestine stores numerous microbial metabolites. Insulin inhibits intestinal lipoprotein secretion and gluconeogenesis. In the intestine, succinate produced by bacteria such as *Anaerobiospirillum succiniciproducens*, *Actinobacillus succinogenes*, *E. coli*, *Corynebacterium glutamicum*, *Mannheimia succiniciproducens*, *Parabacteroides* and propionate constitute a gluconeogenic substrate that stimulates gluconeogenesis as a result of the activation of G6Pase. In turn, butyrate elevates the levels of cyclic adenosine monophosphate (cAMP), which increases gluconeogenic gene expression and elevates gluconeogenesis. Elevated intestinal gluconeogenesis inhibits gluconeogenesis in the liver. Thus, metabolites produced by the gut microbiome participate in glucose and lipid metabolic pathways and energy expenditure.

Gut dysbiosis induces alterations in intestinal metabolites that lead to the development of insulin resistance by influencing insulin-sensitive organs and tissues [98].

**Figure 2:**
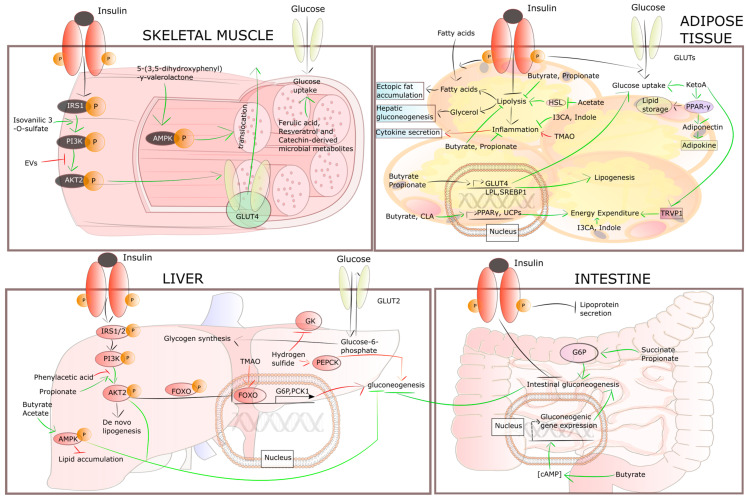
Microbial-derived metabolites and their impact on pathways associated with insulin resistance. Stimulatory interactions are expressed by arrows, and suppression by T-bars. Interactions that promote insulin resistance are expressed in red, while interactions that prevent insulin resistance are expressed in green.

The Gram-negative bacteria, especially *Pseudomonas aeruginosa*, *Helicobacter pylori* and *Salmonella typhimurium* and Gram-positive bacteria, like *Staphylococcus aureus*, *Bacillus subtilis* and *Bacillus anthracis*, form extracellular vesicles (EVs) containing numerous metabolites that favor intracellular pathways promoting insulin resistance. In contrast, other species of *Firmicutes*, *Bacteroidetes*, *Actinobacteria*, *Proteobacteria*, *Pseudomonas* and *Bacillus* produce metabolites such as isovanillic acid, ferulic acid and indole, which increase insulin sensitivity. The mechanisms that link microbiome-derived metabolites and insulin resistance are presented in Figure 2.

Physiologically, in the liver, insulin stimulates de novo lipogenesis and synthesis of glycogen while inhibiting gluconeogenesis by IRS-PI3K-AKT signaling. Propionate elevates both adenosine monophosphate (AMP)-activated protein kinase (AMPK) and AKT phosphorylation, which inhibit gluconeogenesis. The opposite effect is obtained by hydrogen sulfide produced by sulfate-reducing bacteria such as *Desulfobulbus*, *Desulfobacter*, *Desulfovibrio* and *Desulfomonas*; it activates gluconeogenesis by stimulating phosphoenolpyruvate carboxykinase and decreases the synthesis of glycogen by the reduction in glucokinase activity.

A similar effect on gluconeogenesis has been demonstrated by trimethylamine N-oxide (TMAO) released by gut bacteria like *Firmicutes* and *Proteobacteria* from dietary nutrients enriched in choline and carnitine. TMAO elevates gluconeogenesis by the protein kinase R (PKR)-like ER kinase (PERK)-Forkhead box protein O1 (FOXO1) pathway. It activates PERK, which in turn induces the FOXO transcription factor known as a key driver of metabolic diseases. FOXO promotes gluconeogenic enzymes including glucose 6-phosphatase (G6P) and phosphoenolpyruvate carboxykinase 1 (PCK1). Phenylacetic acid, produced by *Bacteroides* spp. and derived from aromatic compounds, decreases AKT phosphorylation. All SCFAs, including acetate, propionate and butyrate—mostly released by anaerobic bacteria such as *Eubacterium rectale* and *Faecalibacterium prausnitzii*—stimulate the phosphorylation of AMPK, which contributes to the inhibition of lipid accumulation [98].

In skeletal muscle, high glucose levels force insulin to activate insulin receptor substrate (IRS)-phosphoinositide-3-kinase (PI3K)-AKT signaling, which in turn leads to uptake of glucose via translocation of glucose transporter 4 (GLUT4). A phenolic metabolite derived from berries, isovanillic 3-O-sulfate, influences the gut microbiota to increase the uptake of glucose via the stimulation of the insulin-mediated PI3K-AKT pathway. Also, 5-(3,5-dihydroxyphenyl-γ-valerolactone produced by *Clostridia*, *Megasphaera massiliensis* stimulates the phosphorylation of AMPK, leading to an increase in GLUT4 translocation to the cell membrane. It has also been observed that resveratrol, ferulic acid and catechin-originated microbial metabolites such as gamma-valerolactones and hippuric acids produced by fecal bacteria, i.e., *Escherichia coli*, also stimulate the uptake of glucose; however, the mechanisms responsible for their actions are still unknown. On the contrary, EV derived from intestinal bacteria inhibit insulin-dependent AKT phosphorylation, resulting in reduced glucose uptake [98].

In adipose tissue, high blood glucose levels induce insulin release, activate both fatty acid and glucose uptake, and inhibit lipolysis. However, disturbances in the inhibition of lipolysis in insulin-resistant adipose tissue results in the elevation of circulating glycerol and free fatty acids (FFAs); this in turn increases the accumulation of ectopic fat in the muscle and liver, and activates hepatic gluconeogenesis. 10-oxo-12(Z)-octadecenoic acid (KetoA), a linoleic acid-derived fatty acid generated by gut lactic acid bacteria, such as *Lactobacilli* and *Lactococci*, elevates insulin-related uptake of glucose and energy expenditure by the activation of transient receptor potential cation-channel subfamily V member 2 (TRPV2). KetoA also enhances both production and secretion of adiponectin by peroxisome proliferator-activated receptor-γ (PPAR-γ) stimulation. Adiponectin is an insulin-sensitizing and anti-inflammatory protein hormone. In turn, TMAO reinforces inflammation in adipocytes by increasing the levels of pro-inflammatory cytokines, especially monocyte chemoattractant protein-1 (MCP-1) and reducing anti-inflammatory cytokines, i.e., interleukin-10 (IL-10) mRNA levels.

In turn, indole and I3CA produced by Gram-positive and Gram-negative bacteria especially *Firmicutes*, *Bacteroidetes*, *Actinobacteria*, *Proteobacteria*, *Pseudomonas* and *Bacillus* present anti-inflammatory activities, elevate energy expenditure and improve insulin sensitivity. Generated by *Propionibacterium*, *Bifidobacterium* and some lactic acid bacteria (i.e., *Lactobacillus plantarum*), conjugated linoleic acid (CLA) stimulates energy expenditure via upregulation of uncoupling protein (UCP) genes in adipose tissue.

All SCFAs decrease lipolysis. Acetate suppresses hormone-sensitive lipase (HSL). Propionate reduces the mRNA expression and secretion of inflammatory cytokines and elevates the mRNA expression of genes involved in lipogenesis, i.e., lipoprotein lipase (LPL), sterol regulatory element-binding protein-1c (SREBP1c) and glucose uptake, i.e., GLUT4. Similarly to propionate, butyrate also suppresses both lipolysis and inflammatory responses. Propionate elevates the uptake of glucose via stimulating GLUT4 expression. In turn, butyrate promotes expenditure of energy via increased expression of PPAR-γ coactivator 1 and UCP genes [98].

To conclude, it is extremely important to maintain the homeostasis of the intestinal microbiome, because it directly affects the type and amount of produced metabolites. The latter, in turn, influence the pathways and biochemical transformations in tissues and organs sensitive to insulin, such as the liver, muscle and adipose tissue. Therefore, intestinal dysbiosis is one of the key factors contributing to the development of insulin resistance. The effect of gut microbiome-derived metabolites on insulin-sensitive cells is depicted in Table 1. Quantitative and qualitative changes in the microbiome, including microbial-derived metabolites, can cause insulin resistance, leading to serious metabolic and physiological consequences and increasing the risk development of life-threatening diseases such as diabetes, obesity, autoimmune diseases and cancer.

## 5. Metformin: Historical Perspectives, Mechanisms, and Gut Interactions

Metformin (a biguanide derivative), is a well-known drug for T2DM treatment. Its history dates back to the Middle Ages, when *Galega officinalis* was used to treat the symptoms of the disease now known as diabetes. *Galega officinalis* is a rich source of galegine, whose glucose-lowering effect was observed in 1917 in experiments on rabbits [126]. After documenting the antihyperglycemic properties of galegine, its synthesis as a drug began in 1920 [127]. Nowadays, it is widely recognized that metformin is an effective, safe, well-tolerated, cheap and globally-available medication which does not cause hypoglycemia if used in monotherapy at an appropriate dose. The multidirectional, beneficial mechanism of action of the drug makes metformin the most frequently prescribed drug not only for the treatment of T2DM, but also for the prediabetes, polycystic ovary syndrome [127,128].

After absorption in the intestine, metformin is distributed to the liver, pancreas, adrenal glands and kidneys, at an approximately sevenfold higher concentration than the serum [4,129]. Finally, the major target organ of metformin is the liver [130,131] where the drug inhibits gluconeogenesis [132,133,134]. The key molecular mechanism of metformin contributing to its glucose-lowering effect is inhibition of hepatic gluconeogenesis via AMPK-dependent and independent mechanisms [135,136,137]. AMPK activation stems from a mild and transient inhibition of complex I (CI) of the mitochondrial electron transport chain (ETC), leading to reduced energy production and a decreased ATP level. This results in a reduction in adenosine triphosphate (ATP) levels and an increase in adenosine diphosphate (ADP) and AMP. Changing the ratio of these three nucleotides leads directly to the activation of AMPK [138]. AMPK, as an intracellular energy sensor, restores energy homeostasis, suppresses anabolic processes, and promotes catabolic processes, thus increasing the rate of glycolysis and reducing the synthesis of nucleic acids, lipids and proteins. During glycolysis, glucose is metabolized to pyruvate, whereas during gluconeogenesis, pyruvate is converted into glucose. In addition, metformin also improves insulin signaling, leading to a subsequent increase of skeletal myocyte glucose uptake [139]. In turn, the AMPK-independent mechanism is associated with metformin-dependent increased redox state in the cytosol, as a result of suppression of glycerol-3-phosphate dehydrogenase 2 (GPD2) activity in the liver. GPD2 inhibition reduces gluconeogenesis, in which redox-originated substrates such as glycerol and lactate are engaged [6].

In addition to the glucose-lowering effect exerted by metformin, growing evidence indicates that the drug decreases the risk of cardiovascular diseases [140,141], inflammation-related diseases [142], cancer [2,143,144,145], obesity, and neurological diseases, as well as various others [146]. Moreover, some researchers suggest that metformin may slow down the aging process [147,148]. Interestingly, some data suggest that the addition of metformin to therapy may suppress or even delay the progression of neurodegenerative diseases, metabolic syndrome, polycystic ovary syndrome, obesity, non-alcoholic fatty liver diseases, metabolic disturbances related to non-retroviral infection diseases, some types of cancers, and atherosclerotic cardiovascular diseases [126]. However, despite clinical observations demonstrating the pleiotropic effect of metformin, its main clinical use is the treatment of T2DM [2].

Surprisingly enough, recent findings suggest that the gut, not the liver, is the principal target of metformin action, and that the intestines play a key role in the regulation of glucose metabolism by metformin. Liu et al. have demonstrated that intravenously administered medicine did not demonstrate any glucose-lowering effects [12,149]. Moreover, the concentration of metformin in the gut was up to 2000 μmol/kg of tissue, i.e., 30–300 times higher than in the plasma [150,151,152]. Gut biopsy performed before and after metformin administration found that the gastrointestinal tract is the key target of the drug [150]. The enterocytes of the gut express organic cation transporter 1 (OCT1), which is responsible for metformin absorption [153]. Dujic et al. report that poorer OCT1 function in patients treated with metformin resulted in high drug concentrations in the intestine, increasing the risk of adverse gastrointestinal (GI) drug reactions [154]. Literature data indicate that approximately 20% of patients taking metformin experience side effects from the gastrointestinal tract, such as diarrhea, vomiting, nausea, and flatulence. Other metformin-induced adverse events such as vitamin B12 malabsorption syndrome and lactic acidosis occur very rarely, and only 5% of patients discontinue treatment with metformin due to the occurrence of GI side effects [2,155,156,157,158,159]. However, the potential mechanisms that are responsible for these side effects are not fully understood. It is believed that intolerance of the drug may be associated with elevated production of lactate [151], the accumulation of serotonin [160], bile acids [161], histamine [162] and the occurrence of polymorphisms in the *OCT1* gene [154,163]. It has also been proposed that the effect of metformin on the gut microbiota may be at least partially responsible for the increased incidence of GI adverse events [23].

A meta-analysis conducted by Szymczak-Pajor et al. found that T2DM treated with metformin and probiotics containing *Clostridium butyricum*, *Streptococcus fecalis*, *Lactobacillus sporagnes*, *Bifidobacterium Bifidum* G9-1, *Bacillus messentericus*, and with Multistrain probiotic: *Lactobacillus acidophilus* W37, *Bifidobacterium lactis* W51, *Bifidobacte-rium bifidum* W23, *Bifidobacterium lactis* W52, *Lacticaseibacillus casei* W56, *Levilactobacillus brevis* W63, *Ligilactobacillus salivarius* W24, *Lactococcus lactis* W58 and *Lactococcus lactis* W19 reduced the risk of bloating, diarrhea and constipation [23]. It seems that the inclusion of probiotics in metformin therapy not only enhances the therapeutic effect of metformin, but also reduces its effects on the GI.

## 6. Metformin Alters the Composition of the Gut Microbiota

Metformin changes the composition of the gut microflora. A number of alterations in gut microflora biodiversity in patients treated with metformin compared to metformin-free patients and healthy people are listed in Table 2 and Table 3. The effect of metformin on the composition of the gut microbiota is not unidirectional. On the one hand, it has been demonstrated that the drug restores the physiological gut microflora by increasing the abundance of bacteria such as *Firmicutes*, *Proteobacteria*, *Escherichia*, *Enterobacter*, *Akkermansia muciniphila*, *Bifidobacterium adolescentis*, *Prevotellaceae*, *Veillonellaceae*, *Bacteroides*, *Megasphaera*, *Bacteroidales*, *Actinobacteria*, *Coribacteraceae*, *Pelomonas* spp., *Staphylococcus*, *Lactobacillus plantarum*, *Lactobacillus reuteri*, *Catenibacterium*, *Lactobacillus*, *Lactobacillus gasseri*, *Streptococcus mutans*, *Butyrivibrio*, *Veillonellaceae*, *Bifidobacterium bifidum*, *Turicibacteraceae*, *Spirochaetaceae* and *Turicibacter* in T2DM patients, compared to both healthy people and T2DM patients not treated with metformin. Moreover, the drug decreases the abundance of some pathogenic bacteria such as *Alkaliphilus*, *Intestinibacter*, *Klebsiella*, *Clostridium botulinum* in patients suffering from T2DM, compared to both healthy people and T2DM patients not treated with metformin. On the other hand, in T2DM patients treated with metformin, an increase in pathogenic bacteria such as *Acinetobacter*, *Pseudomonas*, *Salmonella*, *Shigella*, *Klebsiella*, *Alcaligenaceae* and *Fusobacterium* was observed, compared to T2DM patients not treated with the drug. Additionally, T2DM patients receiving metformin treatment demonstrated lower abundance of some non-pathogenic bacteria, such as *Ruminococcaceae*, *Barnesiellaceae*, *Clostridiaceae*, *Oscilospira*, *Sutterela* spp., *Clostridium coccoides*, *Clostridium*, *Eubacterium*, *Bacteroides*, *Bacteroides intestinalis*, *Bacteroides dorei*, *Bacteroides fragilis*, *Bacteroides caccae*, *Clostridium*, *Clostridium baijerinckii*, *Roseburia*, *Eubacterium eligens*, *Clostridiales*, *Clostridium celatum*, *Prevotella*, *Atopobium*, and *Actinobacteria*, compared to healthy controls.

## 7. Metformin–Gut Interactions Believed to Be Responsible for Its Glucose-Lowering Effect

Metformin is believed to exert its antihyperglycemic effects through the following proposed mechanisms associated with the biodiversity of intestinal flora: regulating glucose absorption in the intestine, influencing the number of SCFA-producing bacteria, controlling intestinal permeability, exerting an anti-inflammatory effect, and altering bile acid circulation.

### 7.1. Regulation of Glucose Absorption in the Intestine

Several lines of evidence indicate that metformin not only increases GLP-1 secretion, but also upregulates SGLT-1, a major glucose transporter in the upper small intestine. Therefore, metformin affects the SGLT-1-dependent glucose uptake pathway in the upper small intestine [87,169,170]. It has also been observed that metformin elevates the abundance of probiotic bacteria [88,89]. Some studies have shown that changes in the intestinal microbiome caused by probiotics and prebiotics are dependent on changes in GLP-1 secretion [171,172]; this is in line with data on changes in genes related to intestinal glucose metabolism observed in germ-free mice (microbial knockout mouse model) transplanted with microbiota from healthy mice [173]. Bauer et al. attribute the metformin-dependent increase in SGLT-1 expression to the effect of the drug on the microbiome of the upper small intestine [174].

A study examining the transplantation of microbiota from HFD-fed rats treated with metformin to those not treated found *Lactobacillus* to be particularly strongly associated with metformin-dependent upregulation of SGLT-1 expression; metformin treatment resulted in a significant increase in the abundance of *Lactobacillus* [174]. Similarly, it was observed that a short three-day metformin treatment of HFD-fed mice increased abundance of *Lactobacillus sanfranciscensis* and elevated SGLT-1 mRNA expression in the upper small intestine [167]. Rooj et al. also confirmed that *Lactobacillus* is associated with metformin-dependent elevated glucose uptake: increasing the levels of SGLT-1-dependent metabolites produced by these bacteria contributed to increased glucose uptake in Caco-2 cells [175]. Other studies have found incubation of Caco-2 cells with the supernatant from *Lactobacillus* cultures to increase the expression of the *GPR120* gene, and thus *GLP-1* expression, by modulating the glucose-sensing pathway [176,177]. Hence, it appears that *Lactobacillus* participates in the modulation of glucose absorption and may contribute to the improvement of glucose parameters in humans and rodents who are supplemented with bacteria [171]. It also seems that a metformin-dependent increase in the abundance of *Lactobacillus* is key factor for the stimulation of the glucose-sensing pathway in HFD-fed rats [174]. However, the molecular mechanism by which the drug affects the prevalence of this genus is still not fully recognized.

It is also worth noting that the results of human and animal studies focusing on the changes in *Lactobacillus* induced by T2DM and metformin are not fully known. In addition to metformin, Sato et al. report significantly higher abundance of *Lactobacillus* in fecal samples from Japanese T2DM patients treated with sulfonylurea (SU), α-glucosidase inhibitors (α-GI), thiazolidine, dipeptidyl peptidase-4 (DPP-4), GLP-1 receptor agonist and insulin with oral therapy, compared to control subjects [164]. Also, the results of studies on the effect of metformin on glucose absorption in the intestine are inconsistent. On the one hand, Koffert et al. have documented that long-term 26-day administration of the metformin increases uptake of glucose in the colon threefold and in the small intestine twofold [178]. On the other hand, Wu et al. have shown that metformin inhibits the absorption of glucose in the small intestine, after just a short seven-day administration of the drug [17].

The effect of metformin on glucose absorption is associated with other strains and types of bacteria inhabiting the human intestines. Lee and Ko report that an increase in the abundance of *Akkermansia muciniphila* is related to decrease in serum glucose levels in HFD-fed mice treated with metformin. Additionally, a decrease in the abundance of the bacteria *Blautia product*, *Clostridium orbiscindens* and *Allobaculum* sp. *strain ID4* in HFD-fed mice treated with metformin is related to an elevation in the levels of glucose transporter 2 (GLUT2) and peroxisome proliferator-activated receptor alpha (PPAR-α) [179]. *Akkermansia muciniphila* is one of the most abundant single species inhabiting the human intestine; an increase in its abundance may decrease the risk of diabetes, obesity and cardiovascular disease development [180]. Substantial enrichment of *Akkermansia*, especially *Akkermansia muciniphila*, as well as an increased abundance of *Verrumicrobia*, were noted in metformin-treated patients with T2DM, as well as in mouse models of obesity induced by HFD [56,57,77,179,181,182]. Interestingly, HFD-fed mice demonstrated a significant increase in glucose tolerance following treatment with *Akkermansia muciniphila* [77,79]. The same response was noted in HFD-fed germ-free mice transferred with fecal material from metformin-treated mice [56].

Taken together, the results of human and rodent studies indicate that a significant increase in the abundance of the genera *Lactobacillus* and *Akkermansia*, especially *Akkermansia muciniphila*, contribute to improved glucose absorption following metformin treatment. These findings hint at the potential value of approaches targeting the gut microbiota for treating T2DM.

### 7.2. The Regulation of Abundance of SCFA-Producing Bacteria

The first studies showing the beneficial effect of metformin on bacteria producing SCFAs were conducted on rodents [179,183,184,185,186,187,188,189]. An increase in the abundance of *Bacteroides* belonging to the genus *Bacteroidetes* was also observed in HFD-fed mice treated with metformin [179,183,184,187]. Zhang et al. reported that metformin-treated db/db mice, a model of T2DM and obesity, demonstrated a greater increase in SCFAs resulting from greater *Bacteroides* abundance in their feces [183]. Similar results were presented in an in vitro study of the gut microbiome. Hao et al. cultured human gut microbiota in 96-well plates to which metformin was added during the lag, log, and stationary phases; following this, microbiome samples were collected at different time points and analyzed for optical density and metaproteomic function. The obtained results indicated that the addition of metformin in the log phase led to significant decrease in bacterial growth. A pronounced increase in the level of *Bacteroides* and a significant decrease in the level of *Clostridiales* was observed as a result of the addition of metformin in the lag phase. A summary of metaproteomic analysis showed that metformin exerted its greatest effect on the functional profile of the microbiome in the lag phase, and this was weaker in the log phase and weakest in the stationary phase [190].

Some studies note a particular increase the abundance of *Butyricimonas spp*. belonging to *Bacteroidetes* phyla, demonstrated in mice treated with metformin [183,184]. *Butyricimonas* spp. produces butyrate participating in insulin sensitivity [104] and stimulation of intestinal hormones, especially GLP-1 and glucose-dependent insulinotropic peptide (GIP) [191]. Additionally, other studies have demonstrated increased abundance of *Allobaculum* and *Parabacteroides* in metformin-treated mice [77,184,187,192]. *Parabacteroides* produce succinate [193], whereas *Allobaculum* is responsible for the secretion of butyrate [194]. Other SCFA-producing bacteria such as *Lactobacillus*, *Akkermansia*, *Ruminococcus*, *Phascolarctobacterium*, *Blautia*, *Coprococcus* or *Butyricicoccus* have also exhibited elevated abundance in rats and mice treated with metformin [77,179,181,183,184].

It is relatively well documented that intestinal dysbiosis is closely related to changes in SCFA levels in humans [195]. In addition, gut dysbiosis in T2DM has been shown to be associated with a decreased SCFA level [196,197,198,199]. In turn, butyrate, lactate and propionate production by the gut microbiota has been found to be increased in patients treated with metformin [56,200,201], as has an increase in the number of bacteria responsible for the production of SCAFs, especially *Blautia*, *Akkermansia*, *Bifidobacterium*, *Shewanella*, *Lactobacillus*, *Butyrivibrio*, *Megasphaera*, and *Prevotella* [56,57,200].

Studies have shown that metformin increases the number of *Bacteroidetes*; these mainly produce propionate and acetate, and have a protective effect against insulin resistance [63,184,185]. However, a study conducted in Nordic T2DM patients treated with metformin showed metformin to have no effect on the level of SCFAs [202]. It cannot be excluded that these discrepancies result from the influence of ethnicity and diet on the gut biodiversity and hence SCFA concentration.

A study based on a multicountry metagenome dataset including 31 patients with type 1 diabetes mellitus (T1DM), 199 with T2DM and 554 without diabetes from Sweden, Denmark and China, examined the effect of metformin on the level of SCFAs. The study revealed a significant increase in *Subdoligranulum* abundance in patients treated with metformin, and a decrease in *Roseburia* and *Subdoligranulum* in those who were not. *Roseburia* and *Subdoligranulum* are responsible for the production of butyrate. The findings indicate a significant increase in the production of both butyrate and propionate in the patients receiving metformin [200].

Higher concentrations of SCFAs, especially butyrate, were observed by Zhernakova et al. in a study on 24 Dutch patients suffering from T2DM. Among these participants, 15 were treated with metformin [203]. Elsewhere, increased concentrations of propionate and butyrate were noted in the feces of a group of men treated with metformin; interestingly, a joint analysis of both sexes did not show any significant changes in the levels of tested SCFAs between the metformin-treated group and the placebo group [56].

Mueller et al. report higher butyrate and acetate levels after six months of metformin treatment in overweight or obese subjects. However, the effect detected after 6 months was no longer observed after 12 months of further treatment with the drug in study participants. It was also observed that an increase in acetate was related to a reduction in fasting insulin. The results from the whole-genome metagenomic sequencing analysis indicate that metformin alters 62 functional metagenomic pathways, including the starch degradation and acetate production pathways, as well as three glucose metabolism pathways: sucrose degradation IV, glucose and glucose-1-phosphate degradation [204].

In summary, human and animal studies suggest that metformin may exert some of its glucose-lowering effects by increasing the level of SCAFs produced by gut microbiota.

### 7.3. The Reduction in Gut Permeability

It has been documented that metformin elevates intestinal abundance of *Akkermansia muciniphila* [77,179,181,183,184,187,192,205,206,207], with the amount being approximately 3–5% higher in healthy subjects compared to subjects with diabetes [76,208]. Metformin treatment was found to improve glucose tolerance in HFD-fed mice treated with *Akkermansia muciniphila*. Additionally, an increase in *A. muciniphila* abundance was positively correlated with mucin-producing goblet cell count in HFD-fed female mice treated with metformin. The increase in the number of goblet cells was independent of diet and metabolic profile [77]. A thickened mucus layer is an essential barrier for LPS [70,71,73].

A study of the transplantation of fecal microbiota from metformin-treated mice to gut-cleansed mice found that metformin evoked changes in the gut microbiome and increased goblet cell number. It was therefore proposed that metformin may inhibit the Wnt pathway, which regulates the differentiation of intestinal stem cells (iSCs) into goblet cells [189].

Lee et al. [179] and Shin et al. [77] also observed upregulation of *MUC2* and *MUC5* genes in HFD-fed female mice treated with metformin. *MUC2* and *MUC5* are responsible for controlling the level of mucin secretion by goblet cells [179]. Therefore, the fact that metformin treatment correlated with an increase in the numbers of goblet cells producing mucin, a substrate for *Akkermansia muciniphila*, may explain the increase in the abundance of *A. muciniphila* after administration of the drug. However, no such increase in the abundance of *A. muciniphila* was noted by Bauer et al. in male HFD-fed rodents treated with metformin; it is worth noting, however, that this analysis was performed in the upper part of the small intestine, while *A. muciniphila* mainly inhabits the colon and cecum, and that this study was conducted on male rodents rather than female rodents. Therefore, the increase in abundance of *A. muciniphila* induced by metformin may be sex-dependent and occur only in females [174].

Zhou et al. report that metformin protected intestinal barrier function by reducing LPS levels in an HFD-induced mouse model of insulin resistance and obesity [182]. It should be underlined that the metformin-mediated increase in the SCFAs butyrate and acetate also activates AMPK and helps enhance intestinal barrier function. AMPK enhances intestinal barrier function by increasing the number of goblet cells by promoting their differentiation and the secretion of mucus. It has been proposed that metformin-mediated activation of AMPK may participate in the reduction of LPS leakage from the gut. AMPK not only enhances the expression of tight-junction (TJ) proteins, which play a key role in the formation of the intestinal barrier, but also inhibits ROS production, contributing to protection against LPS-induced intestinal barrier dysfunction [4,209,210,211].

It can hence be seen that metformin increases the abundance of *Akkermansia muciniphila*; this is related to greater mucin production, which further reduces HFD-enhanced intestinal permeability. However, to date, the mechanisms by which metformin increases bacterial abundance remain unclear. Therefore, to better understand its effect on gut permeability, future research should more closely examine the effect of metformin on the abundance *of A. muciniphila*.

### 7.4. The Anti-Inflammatory Action Mediated by Gut Microflora and Metformin

Evidence from animal models and clinical trials strongly suggests that subacute or chronic inflammation derived from liver and adipose tissue, i.e., *low-grade inflammation* or *sub-inflammation*, leads to insulin resistance and is associated with metabolic diseases such as T2DM and obesity [212,213,214].

Studies indicate that metformin reduces inflammation by inhibiting pro-inflammatory signaling pathways such as the STAT3 [215] or the nuclear factor kappa-light-chain-enhancer of activated B cells (NF-κB) pathway. The drug suppresses tumor necrosis factor-α (TNF-α)-induced phosphorylation of IKKα/β (inhibitor of nuclear factor kappa-B kinase subunit α/β) or the inhibition of IKKα/β degradation in HUVECs (human umbilical vein endothelial cells) and smooth muscle cells [216,217]. It is also indicated that metformin directly suppresses the secretion of pro-inflammatory cytokines, including interleukin 1β (IL-1β), interleukin-6 (IL-6), interleukin-17 (IL-17) and TNF-α, from adipose tissue, colon tissue, pancreas and liver [189,215,218,219].

It has also been suggested that the anti-inflammatory properties of metformin may at least be partially associated with changes in gut microbiome diversity. Indeed, the presence of *Akkermansia muciniphila*, which increases in response to metformin, exerts an anti-inflammatory effect in the gut [77,220,221].

A study on HFD-fed mice identified a reduction in the number of immune-regulating lymphocytes, known as regulatory T cells (Treg), in the stromal vascular fraction of visceral adipose tissue. Interestingly, the number of Tregs was restored to normal values after combined treatment with metformin and *Akkermansia muciniphila*. It was also noted that such treatment was associated with pronouncedly lower mRNA levels of IL-1β and IL-6 mRNA [77]. The cited studies indicate that metformin, in the presence of an abundance of *Akkermansia muciniphila*, reduces the level of LPS and some pro-inflammatory cytokines [183,184,206,207]. Similar results showing the impact of *Akkermansia muciniphila* on inflammation were also reported in studies on humans; however, although these studies indicate a decrease in number of inflammatory markers, improved insulin sensitivity was observed [222].

Metformin also increases the abundance of *Butyricimonas* and *Bacteroides* [181,183,184,187,190,192,223], whose presence negatively correlates with IL-6 level [184]. It is well known that IL-6 has pro-inflammatory effect and attenuates insulin signaling in adipocytes [224,225,226]. As such, metformin-induced downregulation of IL-6 exerts not only an anti-diabetic effect, but also an anti-inflammatory effect in adipocytes and skeletal muscles. Lee et al. found that decreased expression of IL-1β in adipose tissue of aged obese mice was also associated with an increased abundance of *Butyricimonas* and *Bacteroides* in the gut, and that an increased level of IL-1β correlates with the development of insulin resistance [184].

Other studies have also confirmed that metformin treatment appears to downregulate IL-1β, IL-6 and TNF-α, but the reduced expression of these cytokines correlates with different types of bacteria [188,189,218,227]. Wang et al. found reduced serum levels of TNF-α and IL-6 to be related to increased abundance of *Roseburia* and *Akkermansia*, and the Gram-negative bacterium *Prevotella* in the feces of Otsuka Long–Evans Tokushima Fatty (OLETF) rats (a genetic animal model of T2DM) treated with metformin and *Houttuynia cordata* [227]. In turn, Liu et al. demonstrated that decreased TNF-α and IL-6 mRNA expression was associated with an increase in the abundance of *Sutterella*, *Prevotella*, 02d06, and rc4 in an HFD/streptozotocin-induced T2DM rat model treated with metformin [218].

Ahmadi et al. reported markedly decreased mRNA expression of the inflammatory markers TNF-α, IL-1β and IL-6 in HFD-fed mice treated with metformin. The observed decrease correlated with an increase in abundance of *Lactococcus*, *Ruminococcaceae* and S24_7 and a significant decrease in *Veilonellaceae, Coriobacteriaceae, Dorea, Roseburia, Lactobacillus, Dehalobacterium* and SMB53 [189]. It has also been found that combined metformin and *Phellinus linteus* polysaccharide extract (PLAE) diminished gut dysbiosis in Sprague Dawley rats. Both metformin and PLAE significantly reduced serum levels of TNF-α and IL-6, but pronouncedly elevated the abundance of SCFA-producing bacteria [188]. The suppression of pro-inflammatory cytokines plays a key role in attenuating inflammation.

TLR/NF-κB is believed to play a part in intestinal inflammation [228], with intestinal TLR/NF-κB signaling being downregulated by a combination of berberine and metformin induces in db/db mice [183]. Subsequent research showed reduced phosphorylation of IKKα/β upstream of NF-κB signaling in fat, fructose, and cholesterol (FFC)-fed mice treated with metformin [205]. Numerous studies indicate that metformin increases the abundance of gut bacteria, which in turn interact with the immune response in the host; furthermore, metformin treatment results in a significant increase in the number of *Roseburia interstinalis* colonies in the intestinal tract. The growth of these bacteria is associated with NF-kB inhibition [188,189,227,229,230,231].

In conclusion, the metformin-induced decrease in pro-inflammatory markers, especially TNF-α, IL-6 and IL-1β, is associated with an increase in the abundance of physiological bacteria in the gut microbiota. Metformin partially diminishes the gut dysbiosis coexisting with inflammation, observed in T2DM. However, further research at the molecular level is needed to more precisely identify the pro-inflammatory pathways inhibited by metformin by modification of the gut microflora in patients with T2DM.

### 7.5. Actions of Metformin on Bile Acid Circulation

Wu et al. observed elevated plasma BAs levels but unchanged fecal BAs in diabetic rats receiving metformin for four months, with an increase in the level of bsh secreted by the intestinal microflora noted in the second month. It was also found that glycated hemoglobin negatively correlated with the level of unconjugated BAs. Based on these results, it was suggested that metformin may improve glucose metabolism through the impact on the total serum level of BAs [56].

Other studies indicate that metformin may inhibit BA reabsorption in the distal ileum, contributing to an increase in bile salt level in the colon [232,233]. Metformin has also been found to increase intestinal exposure to BAs by inhibiting BA reabsorption [161,189,234,235]. The effect of metformin on BAs was confirmed in a clinical study by Napolitano et al. on patients with T2DM. The treatment increased the level of glycoursodeoxycholic acid (GUDCA; a glycine-conjugated form of the secondary BAs ursodeoxycholic acid) and suppressed reabsorption of BAs, resulting in longer exposure by the intestine and elevated levels in the feces. It has also been suggested that the effect of metformin on BA may be dependent on the influence of the drug on the composition of the gut microbiota. It was observed that metformin increases the abundance of *Firmicutes* and *Bacteroidetes*. Interestingly, the *Firmicutes* number positively correlated with the alterations in cholic acid and conjugate levels, while the *Bacteroidetes* number demonstrated a negative correlation [233].

The prolonged exposure of the intestine to BAs induced by metformin treatment results in a higher degree of binding to intestinal FXR; therefore, it has also been proposed that the effect of metformin on glucose levels may be related to FXR signaling. BAs-FXR signaling decreases intestinal secretion of GLP-1. As GLP-1 increases insulin secretion from pancreatic B cells, a reduction in GLP-1 levels is associated with poorer control of blood glucose levels. Indeed, FXR inactivation was found to result in elevated GLP-1 secretion and better glucose control. Therefore, inhibition of FXR signaling may be a molecular target for the development of new drugs in the treatment of metabolic diseases [236,237,238].

Additionally, improved glucose absorption and upregulation of GLP-1 have been reported in FXR-deficient mice [237]. In line with these results, several studies have documented improvements in insulin sensitivity and glucose tolerance after inactivation of FXR [239,240,241,242,243]. A study analyzing the gut microbiota in patients newly diagnosed with T2DM and those treated with metformin for three days found that the glucose-lowering effect mediated by metformin is related to its influence on the *Bacteroides fragilis*–GUDCA- intestinal FXR axis. As GUDCA is an FXR antagonist and is deconjugated by the gut microbiome, and metformin reduces the abundance of *Bacteroides fragilis*, it appears that the drug suppresses the deconjugation of GUDCA by the bsh activity of *Bacteroides fragilis*, thus increasing GUDCA levels. Finally, the FXR signaling is inhibited, triggering a decrease in glucose level and maintenance of blood glucose level [167]. In line with these results, Bauer et al. observe that the presence of *Bacteroides fragilis* positively correlates with the level of GUDCA in feces [244].

HFD-fed mice treated with metformin were found to harbor elevated levels of *Lactobacillus sanfrancisensis*. The genus *Lactobacillus* is known for inhibiting FXR and increasing ACSL3 (acyl-CoA synthase long-chain family member 3) expression in the upper intestine, leading to increased lipid sensing. Lipid sensing is related to increased levels of lipids in the small intestine and suppression of food intake and production of glucose [244]. Inhibition of FXR leads to the stimulation of lipogenesis via SRBP-1, which activates the genes involved in lipogenesis, i.e., *FAS*, *ACC* and *SCD* [92]. Bu et al. note that knockdown of *ACSL3* reduced the activity of some lipogenic transcription factors, triggering a decrease in de novo lipogenesis [245]. Thus, upregulation of ACSL3 increases lipogenesis [244].

To summarize, metformin inhibits the reabsorption of BAs by influencing the composition of gut microbiota, especially *Firmicutes* and *Bacteroidetes*. This effect leads to increased intestine exposure to BAs. On the one hand, metformin-related prolonged exposure of intestine to BAs elevates their binding to intestinal FXR. In turn, BA-FXR signaling decreases GLP-1 secretion, reducing insulin secretion and resulting in poorer control of the blood glucose level. Furthermore, metformin also reduces the abundance of *Bacteroides fragilis*, which causes an increase in the level of GUDUCA, which acts as an antagonist of FXR signaling, contributing to glycemia reduction. Moreover, metformin also suppresses intestinal FXR signaling and upregulates ASCL3 expression by increasing the abundance of *Lactobacillus*. As a result of these changes, lipogenesis is activated. Therefore, metformin exerts a bidirectional effect on glucose adsorption through its effects on gut microbiota-derived BAs. Further investigations are necessary to recognize the molecular mechanisms by which metformin affects the microbial-derived BA signaling pathways involved in glucose and lipid metabolism.

## 8. Conclusions

**Figure 3:**
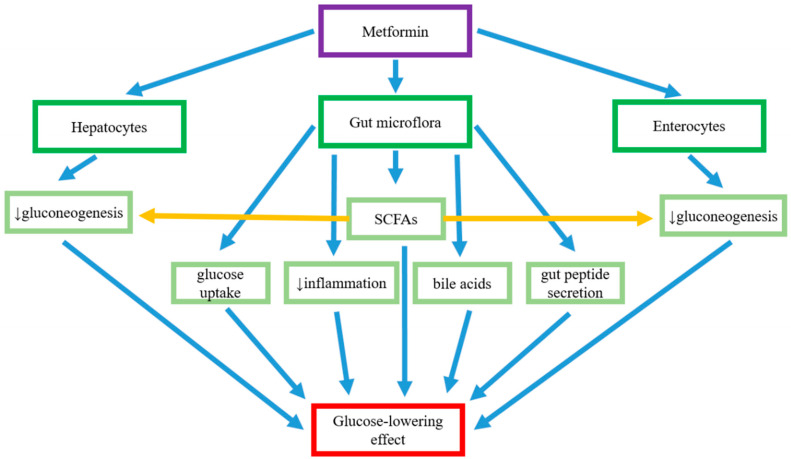
Metformin exerts its antihyperglycemic effects not only by the inhibition of gluconeogenesis in the liver and intestine, but also through partial restoration of the physiological function of gut microbiota. ↓—decrease.

Patients with T2DM often present impoverished biodiversity of the gut microbiota (dysbiosis), whose composition can be influenced by metformin treatment. Indeed, this effect may contribute significantly to the drug’s overall antihyperglycemic action. It can account for the regulation of glucose uptake, increase in SCAF production, maintenance of intestinal barrier function, regulation of bile acid circulation, and the stimulation of the immune response in the gut. The glucose-lowering effect of metformin treatment associated with its effects on the intestinal microbiome are summarized in Figure 3. Metformin partially restores the normal intestinal microflora, and probiotic supplementation brings benefits in patients treated with metformin in the form of a reduction in the occurrence of GI side effects. Therefore, it would be reasonable to undertake further research in people treated with metformin and probiotics, to recognize the mechanisms of this beneficial effect.

## Figures and Tables

**Table 1:** The effect of metabolites produced by gut microbiota on insulin-sensitive cells.

Metabolite (Bacterial Source)	Cell Types/Target Organ/Tissue	Effects	Ref.
Acetate [*Bifidobacteria*, *Bacteroidetes* and *Lactobacillus*]	Hepatocytes	Decreased lipogenesis/increased lipid oxidation	[99,100,101,102]
Propionate [*Propionibacterium* sp., *Clostridium* sp., *Megasphaera* sp., *Propionibacterium shermanii*, *Bacteroides species* (i.e., *Bacteroides fragilis* and *Bacteroides eggerthii)*, *Veillonella* species and *Acidaminococcus* species]		Suppressed gluconeogenesis/decreased lipogenesis/increased lipid oxidation	[99,103]
Butyrate [the *Clostridium* cluster of the phylum *Firmicutes* i.e., *Eubacterium*, *Subdoligranulum*, *Faecalibacterium*, *Coprococcus*, *Anaerostipes*, *Roseburia* and *Anaerobutyricum; Butyricimonas* spp., *Allobaculum*, *Subdoligranulum*]		Decreased lipogenesis/increased lipid oxidaion	[99,104,105]
Hydrogen sulfide [*Desulfobulbus*, *Desulfobacter*, *Desulfovibrio* and *Desulfomonas*]		Stimulated gluconeogenesis/decreased glucogen synthesis	[106]
Phenylacetic acid [*Bacteroides* spp.]		Increased lipogenesis (induced the accumulation of hepatic triglycerides)	[107]
Trimethylamine N-oxide (TMAO) [*Firmicutes* and *Proteobacteria*]		Increased gluconeogenic gene expression (increased gluconeogenesis)	[108,109]
Acetate [*Bifidobacteria*, *Bacteroidetes* and *Lactobacillus*]	Myocytes	Increased lipid oxidation	[110]
Butyrate [the *Clostridium* cluster of the phylum *Firmicutes* i.e., *Eubacterium*, *Subdoligranulum*, *Faecalibacterium*, *Coprococcus*, *Anaerostipes*, *Roseburia* and *Anaerobutyricum; Butyricimonas* spp., *Allobaculum*, *Subdoligranulum*]		Increased lipid oxidation	[103]
Ferulic acid 4-O-sulfate and Dihydroferulic acid 4-O-sulfate (and Trans-resveratrol 4′-O-glucuro-nide, Trans-resveratol 3-O-sulfate)		Increased glucose uptake	[111]
Isovanillic acid 3-O-sulfate		Increased glucose uptake in myotubes	[111]
4-Hydroxy-5-(3,4,5-trihydroxyphenyl) valeric acid, 4-hydroxy-5-(3,4,5-trihydroxyphenyl)-γ-valerolactone and 5-(3-hydroxyphenyl) valeric acid [*Clostridia*, *Megasphaera massiliensis*]		Promoted 2-deoxy-glucose uptake in myotubes (increased glucose uptake)	[112]
Metabolites derived from extracellular vesicles (EVs) [*Pseudomonas aeruginosa*, *Helicobacter pylori* and *Salmonella typhimurium* and gram-positive bacteria, like *Staphylococcus aureus*, *Bacillus subtilis* and *Bacillus anthracis]*		Impaired glucose uptake by decreasing insulin-dependent GLUT4 translocation	[113]
Acetate [*Bifidobacteria*, *Bacteroidetes* and *Lactobacillus*]	Adipocytes	Stimulated adipogenesis/inhibited lipolysis/increased browning	[59,102,114,115,116]
Propionate [*Propionibacterium* sp., *Clostridium* sp., *Megasphaera* sp., *Propionibacterium shermanii*, *Bacteroides species* (i.e., *Bacteroides fragilis and Bacteroides eggerthii)*, *Veillonella species and Acidaminococcus species*]		Increased adipogenesis/inhibited lipolysis	[114,115]
Butyrate [*Eubacterium*, *Subdoligranulum*, *Faecalibacterium*, *Coprococcus*, *Anaerostipes*, *Roseburia* and *Anaerobutyricum*]		Suppressed lipolysis and inflammatory response/improved inflammation/increased thermogenesis	[104,117,118]
Conjugated linoleic acid (CLA) [*Propionibacterium*, *Bifidobacterium* and some lactic acid bacteria (i.e., *Lactobacillus plantarum*)]		Enhanced energy expenditure (increased UCP genes expression)	[119,120,121]
Indole (and Indole-3-carboxylic acid [I3CA]) [*Firmicutes*, *Bacteroidetes*, *Actinobacteria*, *Proteobacteria*, *Pseudomonas*, *Bacillus*]		Decreased inflammation/improved insulin sensitivity	[122]
10-oxo-12(Z)-Octadecenoic acid (KetoA) [gut lactic acid bacteria such as *Lactobacilli*, *Lactococci*]		Induced adipocyte differentiation/increased the production of adiponectin (induced adipogenesis, increased thermogenesis)	[123,124]
Conjugated linoleic acid (CLA) [*Propionibacterium*, *Bifidobacterium* and some lactic acid bacteria (i.e., *Lactobacillus plantarum*)]		Increased energy expenditure	[119,121]
Trimethylamine N-oxide (TMAO) [*Firmicutes* and *Proteobacteria*]		Increased gluconeogenesis (increased gluconeogenic gene expression)	[108,109]
Propionate [*Propionibacterium* sp., *Clostridium* sp., *Megasphaera* sp., *Propionibacterium shermanii*, *Bacteroides species* (i.e., *Bacteroides fragilis* and *Bacteroides eggerthii)*, *Veillonella species* and *Acidaminococcus species*]	Enterocytes	Promoted intestinal gluconeogenesis	[67]
Butyrate [the *Clostridium* cluster of the phylum *Firmicutes* i.e., *Eubacterium*, *Subdoligranulum*, *Faecalibacterium*, *Coprococcus*, *Anaerostipes*, *Roseburia* and *Anaerobutyricum; Butyricimonas* spp., *Allobaculum*, *Subdoligranulum*]		Promoted gluconeogenesis in enterocytes	[67]
Succinate [*Anaerobiospirillum succiniciproducens*, *Actinobacillus succinogenes*, *E. coli*, *Corynebacterium glutamicum*, *Mannheimia succiniciproducens*, *Parabacteroides*]		Activated intestinal gluconeogenesis/ Improved glucose tolerance and insulin sensitivity	[125]

**Table 2:** Changes in gut microbiome in T2DM patients using metformin vs. patients with untreated T2DM. HbA1c: glycated hemoglobin; FPG: fasting plasma glucose; Met+: metformin treatment; Met−: without metformin treatment; T2DM: type 2 diabetes. The direction of the arrows shows changes in the microbiota caused by metformin. ↑—increase; ↓—decrease; NA—not applicable. Bacteria that are part of the physiological intestinal microbiota are marked in green, while pathogenic bacteria are marked in red.

Population	Gut Microbiota	Biochemical and HbA1c; FPG Alterations	References
Spanish Met+ (n = 22) Met− (n = 18) Sex (M/F) Met+ (n = 8/14) Met− (n = 9/9) Age Met+ (52.6 ± 2.0) Met− (54.9 ± 1.9)	Phylum: *Firmicutes *↑, *Proteobacteria *↑ Genus: *Actinetobacter *↑, *Pseudomonas *↑, *Escherichia *↑, *Enterobacter *↑, *Salmonella *↑, *Alkaliphilus* ↓, *Intestinibacter ↓*, *Klebsiella ↓* Species: *Akkermansia muciniphila* ↑, *Bifidobacterium adolescentis* ↑	Butyrate and propionate (in men) ↑, Plasma bile acids ↑, HbA1c-NA FPG-NA	[56]
Colombian Met+ (n = 14) Met− (n = 14) Sex (M/F) Met+ (n = 9/5) Met− (n = 7/7) Age Met+ (50 ± 10) Met− (44 ± 9)	Phylum: *Firmicutes *↑, Family: *Prevotellaceae *↑, *Veillonellaceae *↑, *Ruminococcaceae ↓*, *Barnesiellaceae ↓*, *Clostridiaceae ↓* Genus: *Prevotella *↑, *Oscilospira ↓*, *Bacteroides *↑, *Megasphaera *↑	NA HbA1c ↓ FPG ↓	[57]
Mexican Met+ (n = 14) Met− (n = 14) Sex (M/F) Met+ (n = 2/12) Met− (n = 7/7) Age Met+ (48.1 ± 4.6) Met− (48.1 ± 4.7)	Order: *Bacteroidales *↑ Phylum: *Proteobacteria* ↑, *Bacteroidetes* ↑, *Actinobacteria* ↑ Family: *Coribacteraceae *↑ Genus: *Sutterela* spp. ↓, *Pelomonas* spp. ↑	SCFA production ↑, Gut peptides production, HbA1c ↓ FPG ↓	[58]
Japanese Met+ (n = 17) Met− (n = 33)	Family: *Enterobacteriaceae* ↑ Genus: *Staphylococcus *↑ Species: *Clostridium coccoides* ↓, *Lactobacillus plantarum* ↑, *Lactobacillus reuteri* ↑	NA HbA1c ↓ FPG ↓	[164]
Swedish Met+ (n = 20) Met− (n = 33)	Family: *Enterobacteriaceae *↑ Genus: *Clostridium* ↓, *Escherichia *↑, *Shigella *↑, *Klebsiella *↑, *Salmonella *↑, *Eubacterium* ↓,	NA HbA1c-NA FPG-NA	[165]
USA Met+ (n = 19) Met− (n = 11) Age Met+ (58.2 ± 4.5) Met− (57.5 ± 6.6)	Phylum: *Firmicutes *↑ Genus: *Parabacteroides *↑, *Catenibacterium *↑ Species: *Bifidobacterium *↑	NA HbA1c ↓ FPG ↓	[166]
Chinese Met+ (n = 22) Met− (n = 22)	Genus: *Bacteroides* ↓ Species: *Bacteroides intestinalis* ↓, *Bacteroides dorei* ↓, *Bacteroides fragilis* ↓, *Bacteroides caccae* ↓	GUDCA ↑, Conjugated Secondary bile acids ↑, HbA1c-NA FPG-NA	[167]

**Table 3:** Changes in gut microbiome in T2DM patients using metformin vs. patients without diabetes. HbA1c: glycated hemoglobin; FPG: fasting plasma glucose; T2DM: type 2 diabetic participants taking metformin; ND: non-diabetic participants The direction of the arrows shows changes in the microbiota caused by metformin. ↑—increase; ↓—decrease; NA—not applicable. Bacteria that are part of the physiological intestinal microbiota are marked in green, while pathogenic bacteria are marked in red.

Population	Gut Microbiome	Biochemical and HbA1c; FPG Alterations	References
Swedish T2DM (n = 53) ND (n = 43) Age T2DM (70.5 ± 0.1) ND (70.3 ± 0.1)	Genus: *Clostridium ↓* Species: *Clostridium botulinum* ↓, *Clostridium baijerinckii* ↓, *Roseburia ↓*, *Eubacterium eligens ↓*, *Lactobacillus*↑, *Lactobacillus gasseri *↑, *Streptococcus mutans *↑	C-peptide ↑ HbA1c-NA FPG-NA	[165]
Colombian T2DM (n = 14) ND (n = 84) Sex (M/F) T2DM (n = 9/5) ND (n = 48/36) Age T2DM (50 ± 10) ND (47 ± 9)	Order: *Clostridiales* ↓ Phylum: *Firmicutes*↑, *Actinobacteria*↑ Genus: *Prevotella*↑, *Bacteroides*↑, *Oscillospira* ↓, *Butyrivibrio*↑, *Megasphaera*↑ Family: *Veillonellaceae*↑ Species: *Bifidobacterium bifidum *↑, *Clostridium celatum* ↓ Class: *Mollicutes*↑	NA HbA1c ↑ FPG ↑	[57]
Mexican T2DM (n = 14) ND (n = 76) Sex (M/F) T2DM (n = 2/12) ND (n = 26/50) Age T2DM (48.1 ± 4.6) ND (48 ± 5.4)	Phylum: *Bacteroidetes*↑, *Proteobacteria*↑ Family: *Alcaligenaceae ↑*	NA HbA1c ↑ FPG ↑	[58]
Japanese T2DM (n = 17) ND (n = 50) Age T2DM (62.5 ± 10.8) ND (60.2 ± 12.9)	Genus: *Prevotella* ↓, *Lactobacillus* ↑ Species: *Clostridium coccoides* ↓, *Lactobacillus plantarum* ↑, *Lactobacillus reuteri* ↑, *Atopobium* ↓	Acetic acid ↓, Propionic acid ↓, Fecal organic acids ↓, HbA1c ↑ FPG ↑	[164]
Chinese T2DM (n = 51) ND (n = 26) Sex (M/F) T2DM (n = 28/23) ND (n = 14/12) Age T2DM (58.1 ± 9.4) ND (56.4 ± 10.6)	Phylum: *Actinobacteria ↓* Family: *Turicibacteraceae* ↑, *Enterobacteriaceae* ↓, *Spirochaetaceae* ↑ Genus: *Fusobacterium* ↑, *Turicibacter* ↑	NA HbA1c ↑ FPG-NA	[168]

## Data Availability

No new data were created or analyzed in this study. Data sharing is not applicable to this article.
